# Improving perinatal mood and anxiety disorders through integrated infant mental health care in obstetrics: evidence from a program evaluation study

**DOI:** 10.1186/s12884-025-08444-9

**Published:** 2025-11-26

**Authors:** Jennifer M. Jester, Charity M. Hoffman, Meriam Issa, Jessica. L. Riggs, Hope O’Neill, Michelle Duprey, Katherine Rosenblum, Nora L. Erickson, Cierra Bengel, Chelsea Fisk, Maria Muzik

**Affiliations:** 1 Department of Psychiatry, University of Michigan, 4250 Plymouth Rd., Ann Arbor, MI 48109 USA; 2 University of Michigan School of Social Work, 1080 S. University, Ann Arbor, MI 48109 USA; 3 Department of Psychology, East Tennessee State University, Johnson City, TN USA; 4 Starfish Family Services, 30000 Hiveley, Inkster, MI 48141 USA; 5 Department of Obstetrics & Gynecology, University of Michigan, L4001 Women’s Hospital, 1500 East Medical Center Drive, Ann Arbor, MI 48109-0276 USA; 6 Department of Pediatrics, University of Michigan, L4001 Women’s Hospital, 1500 East Medical Center Drive, Ann Arbor, MI 48109-0276 USA; 7 Department of Pediatrics, University of Minnesota Medical School, 420 Delaware Street SE, Minneapolis, MN 55455 USA

**Keywords:** Pregnancy, Depression, Anxiety, Postpartum period, Prenatal care, Program evaluation

## Abstract

**Background:**

Depression and anxiety in the perinatal period affect many women and have multiple negative impacts on the mother and baby. The Integrated Infant Mental Health approach embeds a Behavioral Health Consultant (IMH-BHC) who has specialized training in Infant Mental Health into OB/GYN clinics. This manuscript reports a quasi-experimental comparison of two groups of women through pregnancy and the first year postpartum, receiving integrated IMH care versus standard OB care. We hypothesized integrated IMH care patients would show less anxiety and depression across pregnancy and postpartum than comparison patients.

**Methods:**

Using a quasi-experimental design, we compared integrated IMH care patients with standard OB care patients to evaluate the primary outcomes of depression and anxiety symptoms from pregnancy through 12 months postpartum across ten obstetric clinics (seven treatment clinics and three comparison clinics) between 2018 and 2021. Data collection included questionnaires in-person, then over the phone during the COVID-19 pandemic. Regression analysis, using fixed effects models to accommodate differences between clinics, compared changes in number of symptoms over time between treatment and comparison groups. Logistic regression was used for comparing number of participants above clinical cutoffs for anxiety and depression symptoms in late pregnancy and at 12-months postpartum. Piecewise linear modeling was used to examine trajectories of symptoms of anxiety and depression.

**Results:**

During pregnancy, depression scores for the intervention group (*n* = 90) remained constant whereas depression in the comparison group (*n* = 68) increased across the later stage of pregnancy. The slope of change for depression scores across the postpartum year was not significantly different in the two groups. Anxiety symptom trajectories did not differ significantly by group membership during pregnancy or in the postpartum period. The intervention group was less likely to be married, to own their homes, or to have completed schooling beyond high school. More participants in the intervention group identified as Black or non-White. Propensity score weighting achieved equivalence in demographics between intervention and comparison groups.

**Conclusions:**

Our findings suggest possible benefits of the integrated IMH model for maternal wellness, most notably for depression symptoms during late pregnancy in a sample of women with high comorbid risk. Supporting at-risk dyads through programs like integrated IMH care represents a much-needed intervention that may make a meaningful difference in the lives of families.

## Introduction

The perinatal period presents unique physical and mental health challenges, with depression and anxiety among the most significant mental health concerns [1]. Maternal suicide due to postpartum mood disorders is the leading cause of preventable maternal mortality [2]. Untreated perinatal mental illness confers risk for negative outcomes, including lower engagement with prenatal care and higher rates of pregnancy/birth complications [3–5]. Moreover, untreated perinatal mood disorders negatively impact child development [5–8] and increase societal costs due to medical expenses and impaired economic productivity among affected parents [4, 9]. Despite these risks, perinatal mood and anxiety disorders are often undiagnosed, leaving patients under- or untreated [10, 11].

Perinatal depression and anxiety are higher for women with additional risk factors, such as preexisting mental health or substance use disorders [12], history of childhood maltreatment or adult interpersonal violence [13, 14], low social support, or high prenatal stress or poverty [7, 15–17]. Untreated mood/anxiety disorders may hinder parental ability to provide supportive parenting, increasing risk for intergenerational transmission of toxic stress and trauma and subsequent negative child outcomes across neurobiological, socioemotional, and behavioral domains [6, 8, 18, 19]. However, research shows that sensitive parenting and secure infant attachment buffers the effects that parental mental illness has on development (e.g., [6, 20–22]). Supporting relational development among mother-infant dyads and targeting psychiatric symptoms can mitigate risks associated with mood/anxiety disorders and promote positive maternal-child outcomes [23].

The field of Infant Mental Health (IMH) is well-positioned to meet the relational intervention needs of perinatal psychiatry, especially when used within integrated models of care [24–26]. IMH services offer relationship-based intervention for parents and infants from pregnancy through 3 years of age. These services support dyads in developing secure attachment relationships, enhancing parental reflective functioning, and improving caregiving sensitivity – thus promoting maternal and child wellbeing [22, 27, 28]. The Infant Mental Health Model includes developmental guidance, material support, parent-infant psychotherapy, facilitation of healthcare needs of parent and infant, provision of emotional support, social support to the parent, safety/crisis planning, and support around special needs that arise during the course of IMH services.

To increase access to prenatal mental health *and* infant mental health in a one-stop care model, Starfish Family Services, a nonprofit agency in Metro Detroit, partnered with local OB/GYN clinics to establish the Integrated IMH Care Model (“Integrated IMH”). This approach pairs the known benefits of embedding behavioral health clinicians into obstetric care [29] with the relationship-focused, trauma-informed, and family-centric therapeutic approach of IMH [22]. Starfish’s Integrated IMH program embeds Master’s-level Behavioral Health Consultants with specialized training in IMH (IMH-BHCs) [30] into obstetric care settings. The multigenerational approach of the IMH-BHCs addresses the needs of families coping with perinatal mood/anxiety disorders. Integrated IMH services are tailored to perinatal individuals and families.

This integrated IMH model is a highly specialized approach that is distinct from usual obstetrics-mental health care integration such as in the perinatal Collaborative Care Model. [CoCM; 31]. The traditional perinatal CoCM targets less complex patients with diagnoses of mild to moderate depression, anxiety, or alcohol misuse [32], and the role of the CoCM-BHC is predominantly symptom tracking and case management, rather than provision of specialized relationship-focused, trauma-informed, and family-centric psychotherapy such in the Integrated IMH model. For a more complete description of services provided by Integrated IMH BHCs, see [33].

This paper reports an evaluation of the integrated IMH model, conducted by a partnership between University of Michigan and Starfish from 2018 to 2021. Using a quasi-experimental design, we compared perinatal patients in integrated IMH clinics (intervention group) with those receiving standard care (comparison group) to evaluate whether the groups differed in depression and anxiety symptoms from pregnancy through 12 months postpartum (primary outcome). The research question is what is the effect on mental health when pregnant women are exposed to an integrated IMH-BHC in their OB/GYN clinic in comparison to routine prenatal care. We hypothesized that patients in integrated IMH clinics would show less anxiety and depression across pregnancy and postpartum than those receiving standard OB care.

## Methods

Between 2018 and 2021, ten obstetrics clinics in Metro Detroit agreed to participate in this evaluation. All clinics were part of three major health systems serving patients in the region and distributed across Metro Detroit serving urban and suburban patient populations. Seven clinics adopted (or had already adopted prior to study) the embedded IMH-BHCs model (intervention group, *n* = 90 patients (57%)), while three clinics provided the comparison group (*n* = 68 patients (43%)). This selection was self-guided by clinic management. Two of three comparison group clinics but only one of seven intervention group clinics were located suburban. The number of participants seen in each clinic varied widely. In the treatment group, five of the clinics had between five and 10 participants, while the other two clinics had 25 and 26 participants, respectively. Comparison participants received standard treatment, which included variable access to social workers and other mental health professionals. This study was approved through the University of Michigan Institutional Review Board (IRB# HUM00127898, original approval 3/20/2017). The research involving human subjects followed the ethical principles in accordance with the Declaration of Helsinki. Recruitment practices differed between groups. Following in-person or phone contact, IMH-BHCs in intervention clinics shared the eligible individual’s name and contact information with research staff. At comparison sites, research staff identified potential participants through electronic medical record review and recruited them by phone. All participants met eligibility criteria of being ≤ 28 weeks’ gestation at the time of recruitment, *≥* 15 years of age, Medicaid-eligible, and residing in metropolitan Detroit. The integrated clinics had an IMH-BHC in the clinic 2–3 days per week. All IMH-BHCs were trained in IMH home visiting services. However, the care they provided in the integrated setting was variable, based on the need of the patient and their openness to services. This resulted in a range of services provided from single check-ins during OB visits that were comparable to more traditional BHC services (but conducted by someone with specialized IMH training) to more intensive care, including more frequent sessions by the BHC, connection to additional services, referrals to outside resources and traditional IMH-HV services (e.g., psychiatric care, home-based IMH therapy, access to material needs).

The IMH-BHC serves as part of the OB care team, and the integration of IMH training expands services beyond those typical of a BHC in OB care settings. They may attend to case management needs and care coordination within the OB clinic itself. An IMH-BHC uses infant mental health perspectives to center the patient and relationships (both parent-baby and parent-provider/health care system) in patient care. An IMH-BHC uses strengths-based approaches and acknowledges systems of oppression and social determinants of health that impact families while helping to identify mental and behavioral health needs. These can include depression, anxiety, trauma/loss/stress, economic needs (e.g., material hardship), substance use, social support needs (including intimate partner violence or other exposures to violence/lack of safety). True to the infant mental health approach, an IMH-BHC may attend to intergenerational patterns, which may be especially salient to a person during pregnancy or just after birth. Overall the IMH-BHC provider supports and enhances obstetrics care within the medical home where an individual is already receiving services. IMH-BHC services an include assessment/evaluation, brief therapeutic intervention, referral to additional resources, and connection to broader, or more traditional IMH services, including IMH home visiting (IMH-HV).

**Fig. 1:**
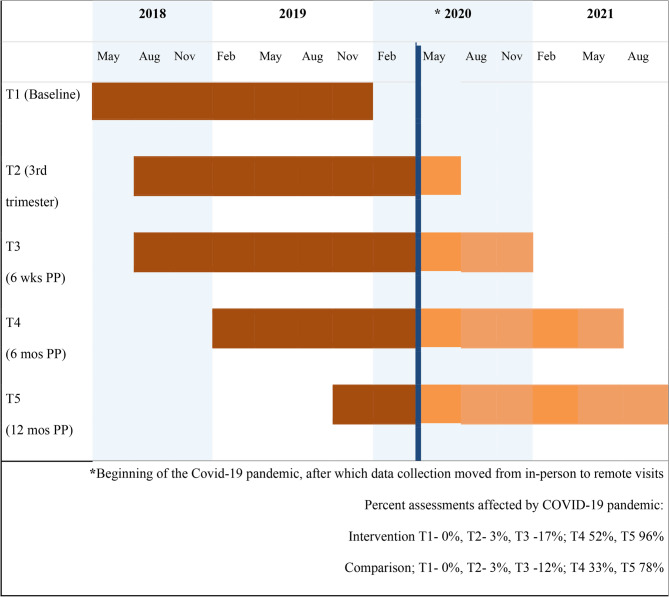
Data collection and Covid-19 timeline

Data collection lasted 30–60 min and included questionnaires about life stressors, trauma, mental health, life satisfaction, use of medical services, and maternal attachment to their child. Prior to the COVID-19 public health crisis, all data were collected in person with a data collector verbally administering questionnaires to participants at their OB/GYN clinic or other location (e.g., library, restaurant, participant’s home). However, COVID-19 restrictions necessitated questionnaires be administered by phone (see Fig. 1 for study timeline). Baseline assessments were completed prior to 28 weeks’ gestation (*n* = 158; 10% first trimester, 77% second trimester, 13% third trimester). Follow-up assessments were completed during the third trimester of pregnancy (28–42 weeks) and three times postpartum (6 weeks, 6 months, 12 months).

### Measures

*Demographics* included maternal race/ethnicity, age, education, and annual household income for the year prior to study enrollment. Participants were asked to report whether they had used substances in the month prior to pregnancy (cigarettes, alcohol, marijuana, or other substance).

*The Protective Factors Survey Social Support scale* [34] measures women’s perception of social support.

*Adverse Childhood Experiences (ACE) Questionnaire* [35] is a 10-item questionnaire that retrospectively assesses traumatic or adverse events (i.e., childhood abuse, neglect, household instability) that occurred to the mother before age 18. Each affirmative answer is given 1 point, with total scores from 0 to 10.

*Patient Health Questionnaire* (PHQ-9; [36]) is a 9-item self-report measure used to screen for symptoms of depression in the last two weeks. Participants rate items on a Likert scale (0, “Never” to 3, “Nearly every day”) with total scores from 0 to 27. Scores of 10 or higher indicates symptoms of moderate to severe depression. Total score is used as a measure of depression symptoms at each time point.

*Generalized Anxiety Disorder Scale* (GAD-7; [37]) is a 7-item self-report measure designed to screen for symptoms of anxiety. Participants rate items on a Likert scale (0, “Never” to 3, “Nearly every day”), and total scores from 0 to 21. Scores of 10 or higher indicate moderate to severe symptoms of generalized anxiety. Total score was used as a measure of anxiety symptoms.

### Data analysis

Using G*power [38], an a priori power analysis, assuming a medium effect size (Cohen’s d = 0.5), power of 0.80, and alpha = 0.05 found a sample size of 64 per group. Therefore, our sample size of 68 in comparison and 90 in intervention group will have a greater than 0.80 power to detect this effect size.

We compared the sample means of symptoms of depression and anxiety, our two primary outcomes, in the last trimester of pregnancy and at 12 months postpartum among participants in the intervention versus the comparison groups. Given baseline differences in demographics and outcomes, we used propensity score analysis to increase equivalence between the intervention and comparison groups [39]. Propensity scoring uses baseline characteristics in a logistic regression model to predict the probability of being a member of the intervention group. From a logistic regression model, we determined that more adverse childhood experiences (ACEs), lower education and income, lower social support, higher likelihood of renting (over homeownership), and higher likelihood of marijuana use in the month prior to pregnancy were predictors of being in the intervention group. Inverse probability weighting was used for subsequent analyses. Weighting increases the contribution from participants who are less likely to receive treatment but actually do receive treatment, while decreasing the contribution of those who do receive treatment and are most likely to receive it. This increases equivalence between groups and provides a weighted estimate of the average treatment effect. Monte Carlo simulation has shown that propensity score methods are valid for sample sizes as low as 40 [39].

We found differential attrition in the repeated measures analysis. Participants with high levels of depression in the intervention group were less likely to drop from the study compared to participants with high levels of depression in the comparison group. To correct for differential attrition, we used logistic regression to predict attrition at the next time point from current variables. The inverse probability of completing the next study assessment was assigned as the attrition weight for that time point [40]. As is common practice, we combined corrections for propensity score and attrition by multiplying the two weights to create a single weight for each participant at each time point [41].

Because participants were seen in different clinics, we sought the best method to account for the clustering. Due to our low number of clinics and variable number of participants within clinics, we chose to use a fixed effects model (FEM) to account for clustering in clinics, which has been shown in simulation studies to be the best choice for this situation [42–44] Fixed effect models are implemented by adding dummy variables for clinics in linear regression models. These models account for the nested structure of the data without explicitly estimating the random effects and require fewer assumptions, which is beneficial in small samples such as ours. We used linear regression FEM to analyze differences in mental health symptoms between treatment and comparison groups.

Missing data. There was substantial attrition in the study (at baseline, *n* = 158, at end of pregnancy, *n* = 134; at 6 weeks postpartum, *n* = 130; at 12 weeks postpartum, *n* = 112 and at one year postpartum, *n* = 96). To address missing data, we used multiple imputation. For the analyses using linear regression, we implemented multiple imputation using “mice” in R. In the imputation model, we included each time point of the mental health symptoms and demographic variables, such as income and education. For piecewise linear modeling, outcome variables at each time point were imputed in SAS, with demographics as auxiliary variables. The imputed data was used in Mplus, using TYPE = IMPUTATION. We repeated analyses with and without multiple imputation to examine the effect of multiple imputation.

We examined changes in mental health symptoms in multiple ways. First, we found the difference in the number of depression and anxiety symptoms from baseline to end of pregnancy and from baseline to the end of the study. As explained above, for these analyses, we used regression with the outcome being the difference in mental health symptoms from baseline to the time of interest, controlling for baseline values of mental health symptoms, education, income and ACE score at baseline for anxiety and depression at end of pregnancy.and dummy variables to account for differences between clinic sites.For the measure of depression at 12 months, we used linear mixed modeling with a random effect of clinic. Second, to control for the confound that the comparison group could receive mental health treatment, we did a t-test comparing those in the treatment group with comparison group participants who had no documented mental health treatment. Finally, to reflect change in slopes of mental health trajectories during pregnancy and postpartum, we used piecewise linear modeling. We defined slopes for the prenatal and postpartum periods using Mplus 8.8 [45], and Wald tests were used to evaluate the difference in slopes between intervention and comparison groups.

## Results

In total, 912 women were screened for recruitment into the study. Of these, 155 were never reached, 386 were ineligible, 213 declined, and 158 were enrolled. Of the 158 enrolled participants, 90 (57%) were intervention patients from 10 clinics with an embedded IMH-BHC and 68 (43%) were comparison group patients from three clinics with no IMH-BHC.

Table 1 shows demographics for participants in the treatment and comparison groups, as well as t-tests comparing the two groups before and after weighting for propensity scores. Prior to weighting, there were substantial differences between the groups; however, propensity score weighting was effective in creating groups which were similar. Table 2 also shows a comparison of clinics features across the treatment and comparison groups.

**Table 1:** *Demographics for intervention and comparison Groups*,* panel A for participants prior to and after Weighting*,* mean (SD); panel B for clinics.* Panel A. Participant descriptives

	Before Weighting				After Weighting			
	Comparison (*n* = 68)	Intervention (*n* = 90)	t	p	Comparison (*n* = 68)	Intervention (*n* = 90)	t	p
Family Income (mean, annual $)	27,090 (16,746)	18,556 (13,434)	3.430	0.001	22,209 (23,809)	20,961 (19,200)	0.510	0.611
Education (mean, years)	13.6 (1.7)	12.4 (1.7)	4.560	0.000	13.0 (2.8)	12.8 (2.2)	0.670	0.506
Total Adverse Childhood Experiences (mean)	2.7 (2.4)	3.9 (3.0)	−2.749	0.007	3.1 (3.8)	3.4 (3.7)	−0.690	0.489
Social Support (mean)	6.2 (1.1)	5.7 (1.5)	2.323	0.022	6.0 (1.8)	6.0 (1.8)	0.220	0.824
Mother Age (years)	28.1 (4.3)	28.7 (6.4)	−0.558	0.578	27.8 (6.6)	29.0 (8.6)	−1.080	0.283
Child Age (years)	1.0 (0.0)	1.0 (0.1)	1.214	0.228	1.0 (0.1)	1.0 (0.1)	1.190	0.236

**Table 2:** *Demographics for intervention and comparison Groups*,* panel A for participants prior to and after Weighting*,* mean (SD); panel B for clinics. *Panel B. Clinic Descriptives

	Comparison Clinics^*^	Intervention Clinics^#^
Location		
Urban	2	3
Suburban	1	4
Individual patients recruited:	68	90
Suburban Clinic location (N, %)	25 (37%)	39 (43%)
Urban Clinic location (N, %)	43 (63%)	51 (56%)

*Panel B. Participating Clinics

^*^Comparison Clinics:

1. Trinity Health IHA Medical Group, Obstetrics & Gynecology – Plymouth (suburban)

2. Henry Ford Medical Center - Ford Road (urban)

3. Henry Ford Medical Center - Detroit Northwest (urban)

^#^Intervention Clinics:

1. Trinity Health IHA Medical Group, Obstetrics & Gynecology – Canton (suburban)

2. Henry Ford Medical Center – Wyandotte Hospital (suburban)

3. Obstetrics & Gynecology Associates - Dearborn Heights (suburban)

4. Beaumont (now Corewell) Health W. Beaumont University Hospital -Royal Oak (suburban)

5. Beaumont (now Corewell) Health Hospital at Monroe Street – Dearborn (urban)

6. Henry Ford Medical Center - Hamtrack (urban)

7. Henry Ford Medical Center - New Center One (urban)

In the intervention group, 65% of patients had any clinic-based in-person encounter with the IMH-BHC. Of these: 70% had one encounter, 17% had 2 encounters, 9% had 3 encounters, 2% had 4 encounters, and 2% had 5 encounters. 35% had phone encounters with the IMH-BHCs. These encounters can result in referrals to other services. None of the comparison participants had encounters with an IMH-BHC during pregnancy. During postpartum, only 12% of women at intervention sites and none at comparison sites had an encounter with the IMH-BHC. For participants in the comparison group, 53 (78%) had no self-reported or chart documented mental health treatment, 8 (11%) had between 1 and 6 visits for mental health treatment and 7 (10%) had between 7 and 56 mental health visits.

**Fig. 2:**
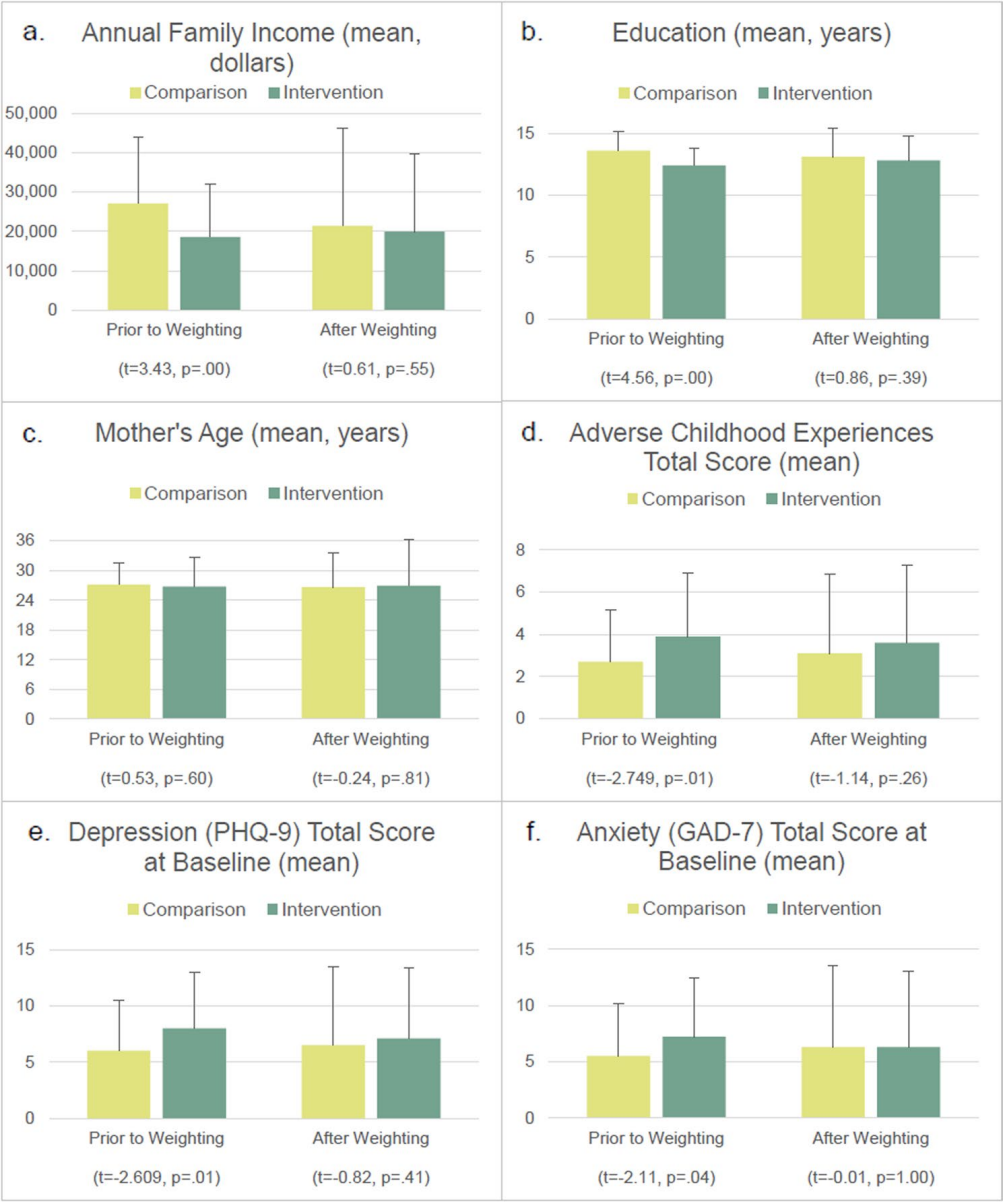
Demographic and clinical characteristics, before and after weighting for propensity for treatment. Note: Comparisons by intervention and comparison groups before and after propensity weighting for (**a**) annual family income in US dollars, (**b**) education, (**c**) age, (**d**) Adverse Childhood Experiences total score, (**e**) depression (PHQ-9) total score at baseline, and (**f**) anxiety (GAD-7) total score at baseline. Comparison n=68, Intervention n=90

Propensity score analysis increased equivalence between the intervention and comparison groups. Fig. 2 shows demographic and clinical characteristics of groups before and after propensity score weighting. Prior to propensity analyses, intervention participants had lower family income, fewer years of education, and higher average scores on ACEs, depression, and anxiety than comparison participants. The average maternal age and race were similar between groups. After propensity weighting, t-tests showed no differences in demographic or clinical characteristics between groups.

Overall, despite relatively high levels of economic need and childhood experiences of abuse or stress, group mean mental health symptoms were reported in the mild range. Only a minority of participants had scores above the cutoff indicating severity suggestive of diagnosis (26% for depression and 27% for anxiety).

**Fig. 3:**
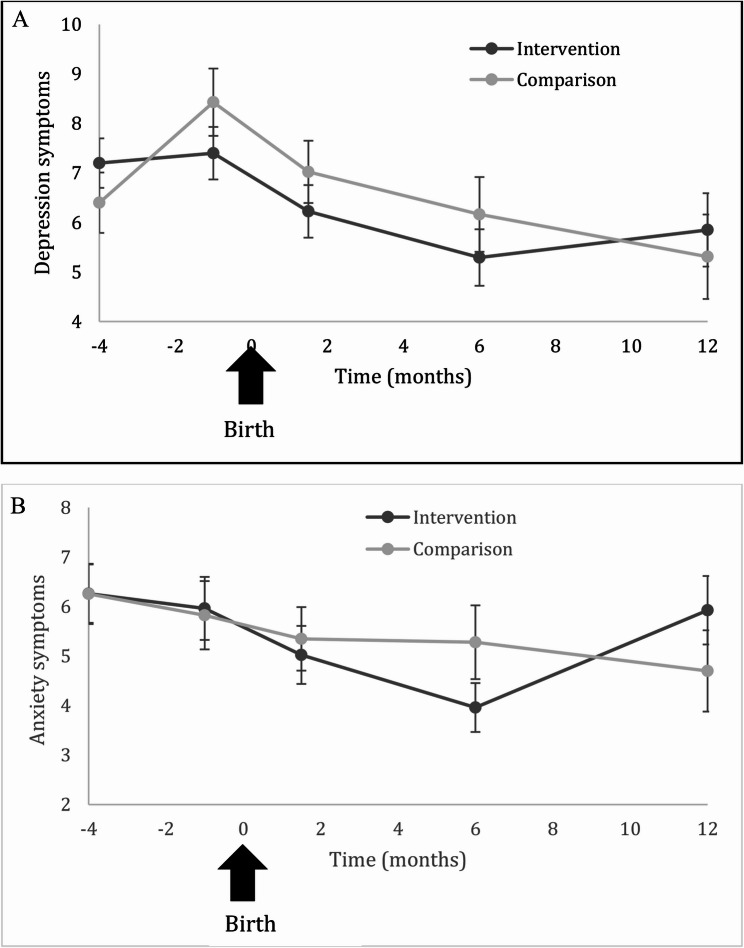
**A** Trajectories of Depression Symptoms (PHQ-9) over Time (means +/- standard error, weighted by propensity scores). **B** Anxiety symptoms over Time (means +/- standard error weighted by propensity scores)

Figure 3A displays means of depression symptoms for the intervention and comparison groups across the study. Intervention participants showed higher levels of depression at baseline than comparison participants. These depression symptoms remained static across pregnancy and showed decreased symptoms in the postpartum period until 6 months postpartum. At 12 months postpartum, the intervention group had a slightly higher mean level of depression than comparison, though intervention group depression scores were still lower at 12-months postpartum than they were at baseline. Comparison participants started with lower levels of depression symptoms at baseline compared to intervention participants; however, their levels of depression increased across pregnancy and were higher than the intervention group during the later prenatal period. Depression scores dropped steadily during the entire first postpartum year but were not lower than the intervention group until 12-months postpartum, despite being lower than the intervention group during initial, baseline assessment.

Regression analysis showed that there were greater changes between treatment and comparison groups from baseline to end of pregnancy for depression when weighting for propensity and attrition, using imputed data (treatment effect = −1.75, SE = 0.81, *p* =.032) or complete data analysis (treatment effect = −1.90, SE = 0.75, *p* =.013)). However, negligible differences between groups were found for anxiety symptoms (with imputed data, treatment effect = −0.55, SE = 1.04, *p* =.60), complete case analysis, treatment effect = −0.70, SE = 0.993, *p* =.48.

For depression changes from baseline to the 1-year postpartum follow-up, linear mixed models showed no treatment effect (estimate − 1.04 (SE = 1.20, *p* =.39) using multiple imputation, and similarly with complete case analysis, treatment effect = −1.56, SE = 1.24, *p* =.21). For anxiety changes from baseline to 1-year postpartum, using multiple imputation, treatment effect = −0.022, SE = 1.34, *p* =.99 and for complete case analysis, the treatment estimate = −0.11, SE = 0.22, *p* =.93.

We used piecewise linear modeling to capture depression trajectories across pregnancy and postpartum. Table 3 displays parameters from the piecewise linear model. Using Wald tests, the prenatal slope of intervention and comparison groups shows significant difference (*p* =.013), whereas the postpartum slopes of intervention and comparison groups do not differ (*p* =.38). This suggests that the comparison group differentially increased in depression symptoms in the prenatal period, compared to the intervention group, but that both groups experienced a similar decrease in depression symptoms across the 12-month postpartum period.

**Table 3:** Piecewise linear model parameters

	Intercept		Prenatal slope		Postpartum slope	
	Estimate [95% CI]	p^a^	Estimate [95% CI]	p^a^	Estimate [95% CI]	p
Depression						
Comparison	6.65 [5.04,8.26]	0.00	0.52 [0.15,0.89]	0.01	−0.460 [−0.70,−0.22]	0.00
Intervention	7.09 [6.05,8.13	0.00	−0.05 [−0.37,0.28]	0.78	−0.280 [−0.46,−0.10]	0.00
Anxiety						
Comparison	6.26 [4.61,7.19]	0.00	−0.33 [−0.74,0.08]	0.11	−0.16 [−0.31,−0.01]	0.03
Intervention	6.25 [5.04,7.46]	0.00	−0.34 [−0.71,0.03]	0.07	−0.15 [−0.38,0.08]	0.20

	Estimate	p^b^	Estimate	p^b^	Estimate	p
Wald Test						
Depression	0.22	0.64	6.20	0.01	0.80	0.38
Anxiety	0.00	0.99	0.00	0.96	0.01	0.91

^a^p value for test of difference from zero

^b^p value for test of difference between intervention and comparison groups

In examining how many women were in a concerning range for depression symptoms, at baseline 20% of intervention and 28% of comparison group women had depression scores above the suggested clinical cutoff (relative risk for intervention condition = 1.39, (Χ^2^(1) = 2.5, *p* =.11). At the end of pregnancy, however, there was a higher rate of depression in the concerning range for the comparison group (38%) vs. the intervention group (27%). Relative risk for clinical range depression score for the intervention group was 0.69, (Χ^2^(1) = 4.70, *p* =.03).

**Fig. 4:**
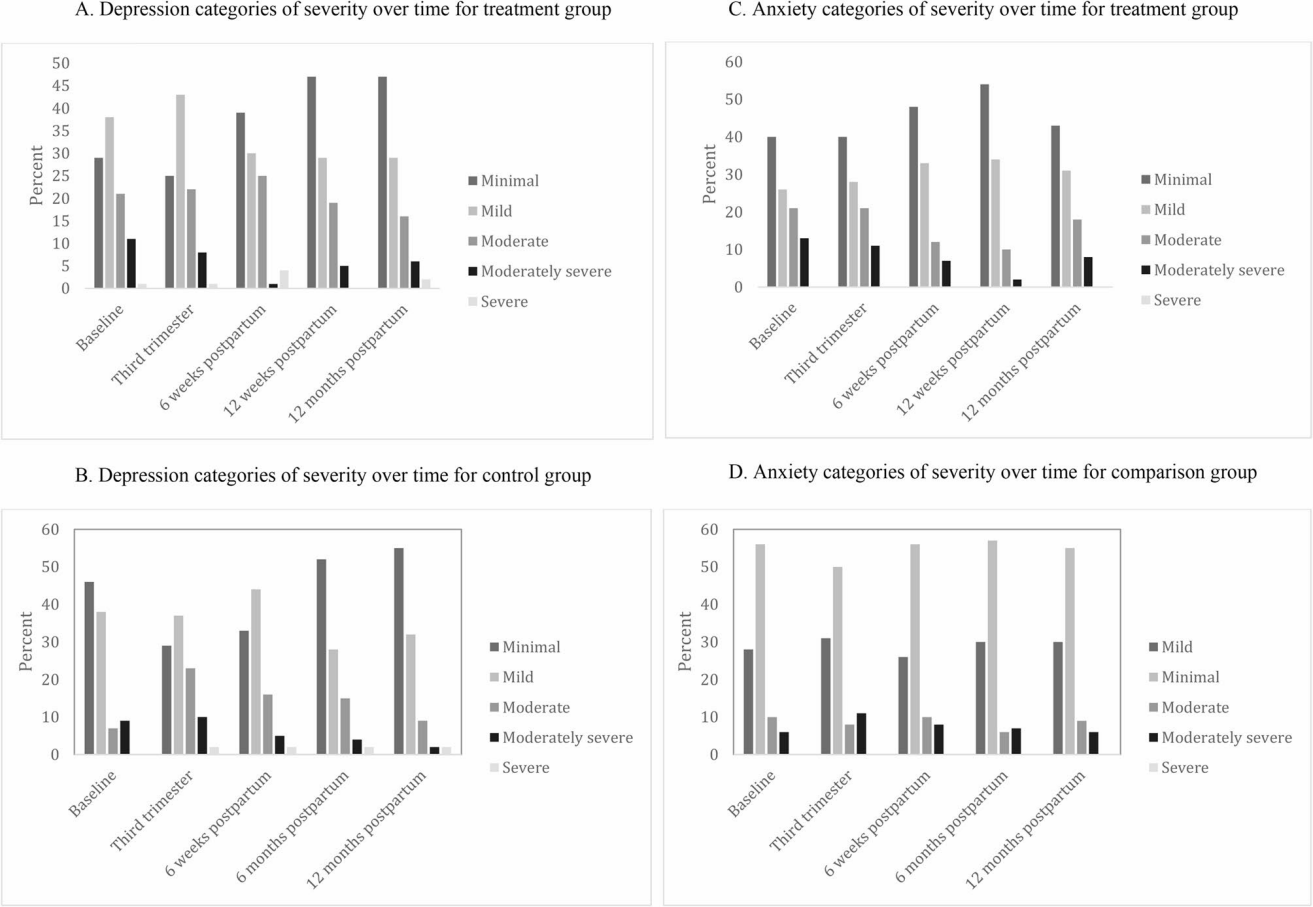
**A** Depression categories of severity over time for treatment group. **B** Depression categories of severity over time for control group. **C** Anxiety categories of severity over time for treatment group. **D** Anxiety categories of severity over time for comparison group

Figures 4A and B show the categories of depression symptoms over time for the treatment and comparison groups, respectively. In Fig. 4B, it is apparent that between baseline and the third trimester, multiple women in the comparison group move into the “Moderate” and “Moderately Severe” categories; this same pattern does not exist in the treatment group as shown in Fig. 4A, where distribution of depression categories is very similar at baseline and in the third trimester. At later points in the postpartum period, more women in the treatment group are in the more severe categories, as expected since they are no longer eligible for treatment and are a higher risk group.

Figure 3B displays anxiety symptoms across the study period in intervention and comparison groups. Both groups decreased in anxiety symptoms across the prenatal period. During postpartum, the intervention group displayed a sharper decrease in anxiety symptoms until 6 months postpartum, and then displayed a rebound in symptoms. In contrast, the comparison group decreased slowly but consistently in anxiety symptoms over the postpartum year. Neither prenatal nor postpartum slopes differed between intervention and comparison groups (*p* =.96 and *p* =.91, respectively, Table 3).

For anxiety, at baseline 29% in intervention group and 23% in comparison group had anxiety scores above the clinical cutoff, a relative risk of 1.27 (Χ^2^(1) = 1.45, *p* =.22). At end of pregnancy, the relative risk was similar, with 27% in intervention group and 21% in comparison group (relative risk = 1.31, Χ^2^(1) = 1.68, *p* =.20). Figs. 4C and D display anxiety by category of severity over time for treatment and comparison groups respectively. For those in the treatment group, the distribution of categories remains relatively constant over time, until 12 months postpartum, where there is an increase in those in “Moderate” and “Moderately Severe” categories (Fig. 4C). Those in the control group show little change in anxiety categories over time (Fig. 4D).

## Discussion

The aim of this study was to compare the effects of an integrated IMH model on depression and anxiety across pregnancy through 12 months postpartum with standard OB care; however, a major world event, the onset of the COVID-19 pandemic, occurred in the middle of assessments for this study. For 85% of participants, the 12-month follow-up occurred between March 20, 2020 and July 26, 2021, the height of the initial COVID-19 pandemic in the Detroit area where the study took place. Because the intervention group was recruited prior to the control group, more intervention participants had assessments during the pandemic than control participants (e.g., 52% of 6-month postpartum assessments for intervention group were during the pandemic vs. 33% for the control group). Moreover, because the intervention group had higher levels of systemic oppression (including lower socioeconomic status and higher levels of childhood trauma), they were likely more adversely affected by the pandemic than the higher-resourced comparison group, in a manner that confounded results in an unknown way. For example, twice as many women in the comparison group than the treatment group were married (28% vs. 14%), twice as many in the comparison group owned their own home (22% vs. 11%). Importantly, the level of education was lower in the treatment group, with more than twice as many participants having high school education or lower (56%) vs. comparison group (32%). In addition, more participants in the treatment group identified as Black (61% vs. 54%) and more participants in the treatment group identified as a race other than White (84% vs. 71%). These measures represent experiences of systemic oppression likely exacerbated by the pandemic [46–48] and complicate interpretation of the data for the postpartum assessments, but do not affect the pregnancy assessments. Therefore, we concentrate our interpretation of results on the earlier assessments. This approach allowed us to utilize data that was impacted by the global pandemic, but still offers valuable insight into health care practices supporting women during a vulnerable time. Indeed, capturing the impact of intervention in the context of real-world stress events can add vital information about health experiences in the context of a pandemic, therefor further adding to the literature.

The results of piecewise linear modeling indicate that, during pregnancy, depression scores remained constant for the intervention group and increased for the comparison group in late pregnancy. For anxiety, there was no group difference in trajectories, and both groups showed a steady decline in symptoms from pregnancy to six months postpartum. At 12 months postpartum, depression and anxiety symptoms were not significantly different in the treatment and comparison groups. This is within the context of overall reported depression and anxiety symptoms being mild to moderate, indicating that many patients receiving IMH-BHC services entered the study with few reported mental health needs. This may have constrained the ability of this study to measure the impact of intervention on reducing depression and anxiety symptoms, with a large proportion of participants demonstrating less need at study outset.

Our findings partially support the maternal wellness benefits of integrated IMH care, most notably in relation to depression symptoms during late pregnancy. Prior work confirms that depressed mood has a bimodal pattern in pregnancy, with greater symptoms in early and late gestation [49], especially among women with comorbid risk factors (i.e., economic adversity, trauma exposure). The women in this study had comorbid adversities, including high rates of childhood abuse and socioeconomic disadvantage – psychosocial factors consistent with an expected increase in 3rd trimester depressive symptoms. However, only the comparison group exhibited this increase in depression symptoms, suggesting that IMH-BHC services may demonstrate a preventive or buffering effect against maternal depression in the prenatal period. Women in the intervention group, though initially reporting higher depression scores earlier in pregnancy, did not have increased depression scores in late gestation. We speculate that access to the IMH-BHC helped participants develop coping strategies to reduce stress, which helped prevent increases in depressive symptoms. This is especially cogent because postpartum depression is a major risk factor for maternal suicide; rates of perinatal suicidality has increased three-fold as of 2017 [2], constituting an urgent public health concern in populations experiencing a number of systemic oppressions.

We regard the reduced late gestational depressive symptoms among at-risk women in the intervention group as highly beneficial to mother and fetus. Prior work has related high levels of prenatal depression to high cortisol in the amniotic fluid and placental barrier, impacting fetal brain development and functionality [2], fetal heart rate [50] and birth outcomes [51, 52]. Untreated perinatal mood/anxiety disorders are correlated with negative fetal and infant outcomes, including premature birth and low birth weight [52, 53], difficult infant temperament, and challenges to mother-infant bonding [54, 55]. Ultimately, infants of mothers with mood or anxiety disorders are at greater risk of developing insecure attachment [19, 56, 57].

In our study, the largest effect on depression symptoms coincided with highest frequency of interactions between participants and the IMH-BHCs. The lower level of depression symptoms in the intervention group was no longer evident at six months postpartum. One possible explanation for this is that the integrated IMH intervention reduced in frequency and consistency during the postpartum year. These findings may suggest the need for sustained support for families across both pregnancy and the postpartum period, possibly through IMH integration across development [58] that bridges obstetric care for the mother with pediatric care for the child and family. In traditional IMH services, which are typically conducted as part of a home visiting psychotherapy service (Infant Mental Health Home Visiting; IMH-HV), services are routinely offered to eligible families through the child’s third birthday. It is logical that any positive impact of IMH-BHC services would be sustained across infancy and early childhood as well.

Future efforts to integrate IMH services into pediatric settings may serve to bridge the transition from care in obstetric/gynecologic settings to pediatric primary care settings. IMH-BHC providers may serve a unique purpose in supporting this transition seamlessly, while holding the entire family in mind, promoting parent mental health, infant/early childhood development, and relational health via integrated care settings.

We speculate that postpartum benefits were offset by the COVID-19 pandemic. In the face of this crisis, it is not surprising that the initial effects of improving mental health were not sustained through the first postpartum year.

For anxiety symptoms, the trajectories for the intervention and comparison groups were similar throughout the study. Both groups showed decreasing anxiety levels across the last months of pregnancy and slightly decreasing levels of anxiety across the first six months postpartum. Anxiety symptoms for the intervention group showed an increase at the 12-month follow-up. Similar to depression symptoms, we hypothesize that this increase is due to the stress from the COVID-19 pandemic.

## Strengths and limitations

A major strength of this study is that it was community-based, and the intervention participants reflected families who are most in need of extra prenatal and postpartum support. An additional strength is that the Behavioral Health Consultants were trained IMH providers, a model which has been shown to be effective in decreasing child abuse potential [59] and increasing maternal sensitivity [22] when applied in the first three years of life. Our utilization of multiple measures of mental health in pregnancy and the first postpartum year allowed us to view trajectories of maternal mental health throughout this critical period. Finally, our propensity score analysis created a quasi-experimental design, which allowed comparison of intervention and comparison groups.

The main limitation of the study was the small sample size and lack of random assignment. Although we used propensity score analysis to make groups more comparable at baseline, a follow-up trial with random assignment of BHCs would help clarify results. In addition, a larger sample size would enable analysis of a cluster randomized trial, with random effects for clinic. In the current small sample study, we used fixed effects to account for clustering within clinics. The results are also confounded to an unknowable extent by the COVID-19 pandemic, which started in the middle of the study. Another limitation is the lack of measurement of mental health early in pregnancy, as well as the fact that we did not collect information on referred resources – both variables which would create a more complete picture of perinatal mental health.

## Conclusion

Pregnancy is often a time of challenges, with epidemiological data confirming increased risks for mood and anxiety disorders. Integrated Infant Mental Health models of care provide access to Behavioral Health Consultants with Infant Mental Health training in OB/GYN clinics to increase access to care and decrease stigma of mental health care. Because our study found that participants in the integrated-IMH clinics demonstrated less of an increase in late gestation depression than the comparison, integrated-IMH care may represent a feasible, accessible intervention at a critical period of development for both mother and baby. Lower levels of depression in this phase can lead to better outcomes for the mother-baby dyad and can lower the risk for intergenerational transmission of trauma. Supporting at-risk mothers with targeted and accessible mental health care during this period – through programs such as integrated-IMH services – has potential to make a meaningful difference in the lives of families. OB/GYN clinics and other practices which routinely care for the mother-baby dyad might consider incorporating integrated-IMH care as a feasible clinical tool for supporting at-risk patients. Additional research is needed to gain a complete picture of depression and anxiety symptoms across pregnancy (i.e., a randomized controlled trial in the community). Furthermore, assessing direct benefits to mother-infant interactions and infant outcomes would strengthen relevance from a relational health standpoint.

## Data Availability

The dataset analyzed during the current study are not publicly available due to privacy considerations but are available from the corresponding author on reasonable request. This study was approved through the University of Michigan Institutional Review Board (IRB# HUM00127898, original approval 3/20/2017).

## Abbreviations

IMH: Infant Mental Health
BHC: Behavioral Health Consultant
IMH-BHC: Infant Mental Health Behavioral Health Consultant
ACE: Adverse Childhood Experience
PHQ: Patient Health Questionnaire
GAD: Generalized Anxiety Disorder
OB: Obstetrics
